# Release of ballast material during sea-ice melt enhances carbon export in the Arctic Ocean

**DOI:** 10.1093/pnasnexus/pgae081

**Published:** 2024-02-17

**Authors:** Steffen Swoboda, Thomas Krumpen, Eva-Maria Nöthig, Katja Metfies, Simon Ramondenc, Jutta Wollenburg, Kirsten Fahl, Ilka Peeken, Morten Iversen

**Affiliations:** MARUM—Center for Marine Environmental Sciences, University of Bremen, 28359 Bremen, Germany; Alfred Wegener Institute, Alfred Wegener Institute Helmholtz Centre for Polar and Marine Research, 27570 Bremerhaven, Germany; Alfred Wegener Institute, Alfred Wegener Institute Helmholtz Centre for Polar and Marine Research, 27570 Bremerhaven, Germany; Alfred Wegener Institute, Alfred Wegener Institute Helmholtz Centre for Polar and Marine Research, 27570 Bremerhaven, Germany; MARUM—Center for Marine Environmental Sciences, University of Bremen, 28359 Bremen, Germany; Alfred Wegener Institute, Alfred Wegener Institute Helmholtz Centre for Polar and Marine Research, 27570 Bremerhaven, Germany; Alfred Wegener Institute, Alfred Wegener Institute Helmholtz Centre for Polar and Marine Research, 27570 Bremerhaven, Germany; Alfred Wegener Institute, Alfred Wegener Institute Helmholtz Centre for Polar and Marine Research, 27570 Bremerhaven, Germany; Alfred Wegener Institute, Alfred Wegener Institute Helmholtz Centre for Polar and Marine Research, 27570 Bremerhaven, Germany; MARUM—Center for Marine Environmental Sciences, University of Bremen, 28359 Bremen, Germany; Alfred Wegener Institute, Alfred Wegener Institute Helmholtz Centre for Polar and Marine Research, 27570 Bremerhaven, Germany

## Abstract

Globally, the most intense uptake of anthropogenic carbon dioxide (CO_2_) occurs in the Atlantic north of 50°N, and it has been predicted that atmospheric CO_2_ sequestration in the Arctic Ocean will increase as a result of ice-melt and increased primary production. However, little is known about the impact of pan-Arctic sea-ice decline on carbon export processes. We investigated the potential ballasting effect of sea-ice derived material on settling aggregates and carbon export in the Fram Strait by combining 13 years of vertical flux measurements with benthic eDNA analysis, laboratory experiments, and tracked sea-ice distributions. We show that melting sea-ice in the Fram Strait releases cryogenic gypsum and terrigenous material, which ballasts sinking organic aggregates. As a result, settling velocities of aggregates increased ≤10-fold, resulting in ≤30% higher carbon export in the vicinity of the melting ice-edge. Cryogenic gypsum is formed in first-year sea-ice, which is predicted to increase as the Arctic is warming. Simultaneously, less sea-ice forms over the Arctic shelves, which is where terrigenous material is incorporated into sea-ice. Supporting this, we found that terrigenous fluxes from melting sea-ice in the Fram Strait decreased by >80% during our time-series. Our study suggests that terrigenous flux will eventually cease when enhanced sea-ice melt disrupts trans-Arctic sea-ice transport and thus, limit terrigenous-ballasted carbon flux. However, the predicted increase in Arctic primary production and gypsum formation may enhance gypsum-ballasted carbon flux and compensate for lowered terrigenous fluxes. It is thus unclear if sea-ice loss will reduce carbon export in the Arctic Ocean.

**Significance Statement**

The biological pump drives the export of organic matter from the surface to the deep ocean, mediating carbon sequestration and regulating the global climate. Climate change induced warming in the Arctic leads to rapid sea-ice decline; however, the consequences for carbon export are not well understood. Here, we show that ice-rafted terrigenous and cryogenic minerals within the sea-ice are released into the surface water during sea-ice melt and can ballast prevailing organic particles. This leads to an increase in particle settling velocity, thus strengthening carbon export via the biological pump. With the continuous loss of sea-ice, fewer terrigenous ballasting minerals are released into the water column, while sea-ice-associated primary production and ballasting via cryogenic minerals are predicted to increase.

## Introduction

The biological pump drives the export of organic matter from the surface to the deep ocean, mediating carbon sequestration and delivering organic matter to subsurface and benthic ecosystems (1, 2). The main vectors for organic matter export are zooplankton fecal pellets and marine snow particles which are composed of the prevailing material in the water column, including inorganics (e.g. minerals), living, and/or detrital organic material (3). The efficiency with which marine snow particles and zooplankton fecal pellets are transferred to the deep ocean is largely determined by their settling velocity and the rate at which the contained organic matter is degraded. The most common degradation mechanisms are zooplankton grazing and microbial remineralization (4). Generally, 90% of flux attenuation occurs in the upper 100 m of the water column where zooplankton abundance and biological activity are highest (4, 5). Therefore, fast-settling particles are more likely to escape degradation through the water column and reach the deep ocean and sediments. As a result, particle settling velocities are a controlling factor for the extent of particle degradation, i.e. the time available for degradation, in the water column (4).

In the sea-ice covered Arctic, brine channels in the ice (6), ice ridges, the snow-ice interface, and under-ice areas act as a habitat for ice-, cryopelagic-, and under-ice algae (7, 8). In addition, sea-ice melt induces surface stratification which promotes the formation of phytoplankton blooms that form long narrow belts along the ice edge (9, 10). The diverse phytoplankton communities associated with sea-ice may therefore sustain enhanced primary production and have been observed to increase flux of particulate organic carbon (POC) in the vicinity of the ice edge compared to ice-free areas (11–14). This enhanced POC flux was explained by increased sinking velocities in the sea-ice vicinity (14, 15).

Increased particle settling velocities can be caused by the incorporation of ballasting components (e.g. sediments or minerals) into marine particles (4, 16–18). Recently, cryogenic gypsum was discovered as a sea-ice mediated ballasting component in the Arctic Ocean (19). Cryogenic gypsum crystals form during ion precipitation in sea-ice brine (20) and were observed to be released during ice melt (19, 21). The released cryogenic gypsum may ballast marine particles and aggregates in the water column, including slow-sinking colonies of *Phaeocystis* spp., causing them to sink faster (19). While it was estimated from model predictions that gypsum precipitation may amount to 2.7 g m^−2^ (19), it is so far unclear to which extent gypsum is released to the water-column, and thus their ballasting potential is not fully exploited yet.

In addition to cryogenic gypsum, Arctic sea-ice can contain large amounts of terrigenous material (22), which can also serve as a ballasting component. During the formation of sea-ice on the shallow Siberian shelf, terrigenous particles are incorporated by suspension freezing or by anchor ice which plows through shallow sediments (22, 23). Subsequently, the sea-ice is transported across the Arctic Ocean by the Transpolar Drift and the ice-rafted sediments are released during sea-ice melt, which is most intense in the Atlantic sector of the Arctic (24, 25).

Sea-ice rafted sediments are mostly composed of quartz and clay minerals (22, 26, 27), which were observed to efficiently ballast marine snow particles in laboratory experiments, resulting in 2-fold higher particle settling velocities compared to nonballasted particles (17, 22). Typically, terrigenous and POC flux are high in the vicinity of the sea-ice edge (11), and it was suggested that ballasting sediments may drive a more efficient POC export in the Arctic Ocean (28).

The continuous reduction of the Arctic sea-ice extent and the transition from multiyear toward first-year sea-ice may have strong repercussions on sea-ice-mediated particle ballasting. Since only shelf-formed ice contains terrigenous material, the reduced sea-ice formation on the Siberian shelf decreases the release from melting sea-ice (24, 29). Cryogenic gypsum precipitation is not restricted to sea-ice formation in shelf areas and an increasing sea-ice retreat during the summer months yields an increased sea-ice growth in winter (30). As gypsum forms predominantly in newly formed first-year sea ice, this may result in an increased gypsum formation (21). In addition, thinning of sea-ice leads to more light availability within the sea-ice and upper water column, which increases sea-ice and pelagic-associated primary production (31, 32). It is, therefore, unclear if sea-ice ballasting by cryogenic gypsum may compensate for a decrease in terrigenous particle ballasting and potentially drive an enhanced carbon export in a more productive future Arctic Ocean.

**Fig. 1.**
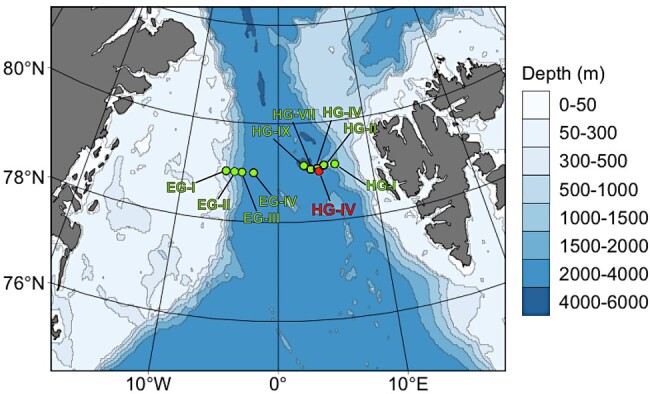
Overview map of the sampling stations located in the LTER observatory HAUSGARTEN in the Fram Strait. The red circle indicates station HG–IV where the sediment trap mooring was deployed. Green circles indicate the benthic sediment sample sites.

In this study, we utilize continuous flux measurements from sediment traps (SMT) at station HG-IV (in the central Fram Strait; Fig. 1) between 2000 and 2013 in combination with eDNA sediment samples, remote sensing data on sea-ice distributions as well as laboratory experiments to assess the role of sea-ice mediated ballasting (33). We focus on the export of the marine haptophyte *Phaeocystis* spp. as model organism, as it is neutrally buoyant and therefore does not contribute considerably to export flux below 100 m water depth if not ballasted (19, 34–37). We assessed the role of the sea-ice edge for carbon export and consequential sympagic-pelago-benthic coupling and suggest that future sea-ice decline may reduce carbon export due to the loss of sediment material, while a predicted increase in first-year sea-ice extent may enhance gypsum ballasting and thus potentially (over)compensate a loss of terrigenous particle ballasting.

## Results

### Sea-ice cover and export fluxes at station HG-IV in the central Fram Strait

**Fig. 2.**
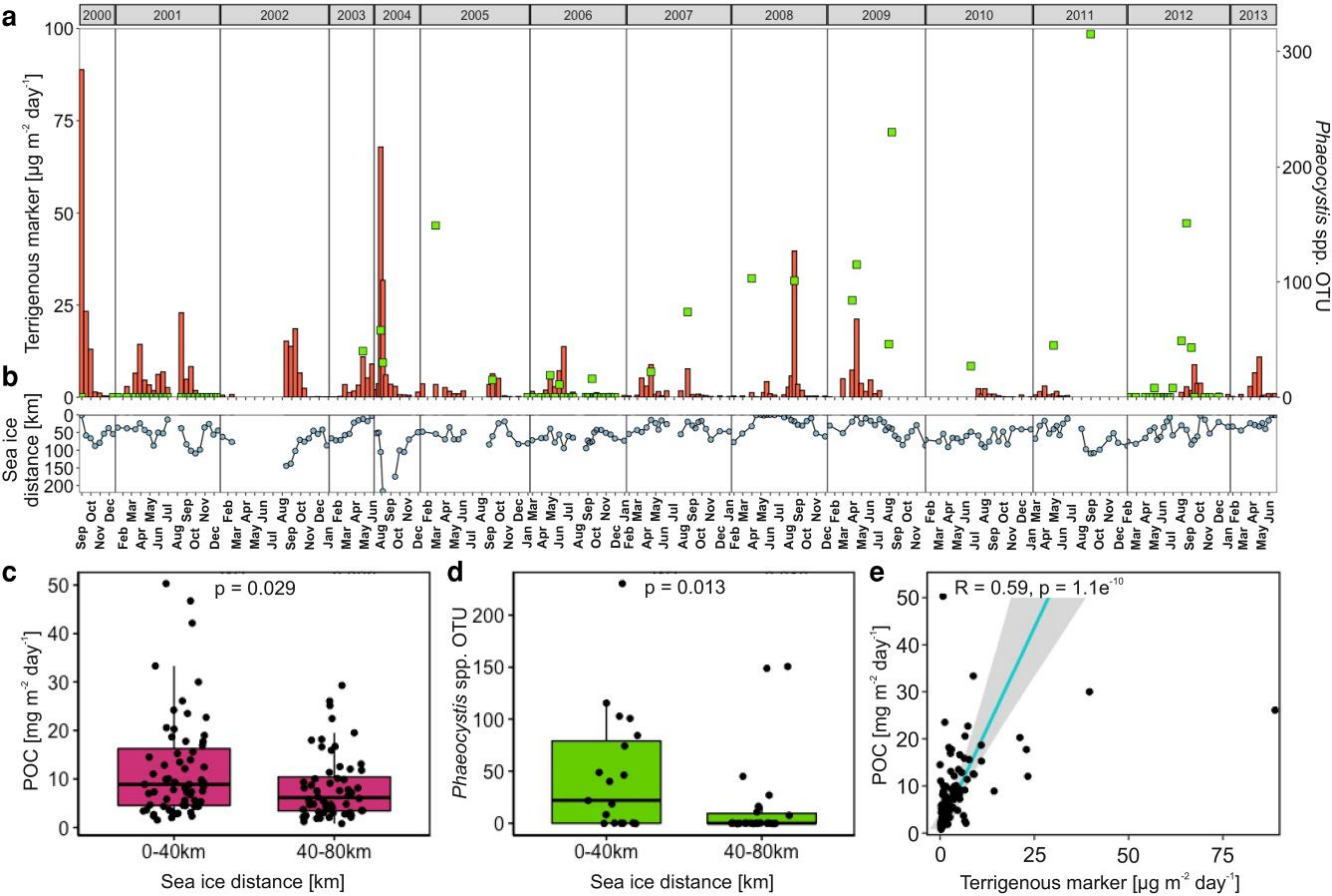
Sediment trap fluxes and sea-ice dynamics measured at the mooring station HG-IV from 2000 to 2013. Panel (a) shows sediment trap mooring fluxes of terrigenous marker (orange bars) and *Phaeocystis* OTUs (green squares). Panel (b) shows the average distance of the sea-ice edge (defined as ≥15% sea-ice cover) to the mooring location. Panels (c) and (d), respectively, show POC flux and sequence abundance of *Phaeocystis* OTUs at HG-IV, grouped according to the distance of the sea-ice edge at the time of individual flux measurements. Panel (e) shows fluxes of POC against terrigenous marker collected at HG-IV (linear regression, gray area shows the 95% confidence interval). No sediment trap was deployed at HG-IV between July 2003 and June 2004 which led to the missing flux measurements in panels (a) and (b) (see Table S1 for details). Due to irregular sampling frequency, the sampling months are stated in panel (a). For panels (c to e) only flux values during the productive season from March to September were used.

**Fig. 3.**
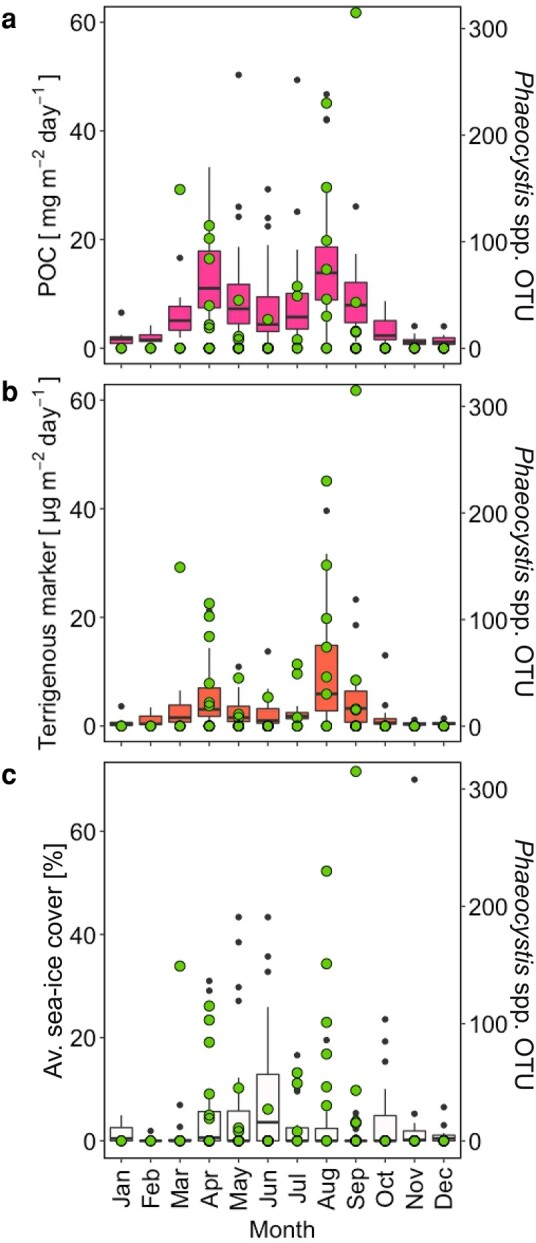
Monthly sediment trap fluxes and sea-ice cover measured at the mooring station HG-IV from 2000 to 2013. Panels (a) and (b) depict the monthly POC and terrigenous marker flux. Panel c shows the monthly average sea-ice cover over the mooring location at HG-IV. Green circles in panels (a–c) show the sequence abundance of *Phaeocystis* OTUs in the sediment trap samples.

Sediment trap fluxes of POC and relative contributions of 18S-sequences of *Phaeocystis* spp. (*Phaeocystis* spp. Operational Taxonomic Units, respectively, *Phaeocystis* spp. OTUs) between March and September were significantly higher when the distance to the ice edge was 0–40 km from the trap compared to POC flux and *Phaeocystis* spp. OTUs were collected while the ice edge was 40–80 km from the trap (Wilcoxon signed-rank test; *P* < 0.05; Fig. 2c and d). POC flux measured when the distance to the ice edge was a 0–40 km showed no trend toward an increase or decrease between 2000 and 2013 (Seasonal Mann–Kendall test; *P* > 0.05). The flux of POC and sterol-based terrigenous markers from March to September were significantly positively correlated (Spearman's rank correlation coefficient; *P* < 0.001; Fig. 2e). The monthly export flux of POC, terrigenous marker, and sequence abundance of *Phaeocystis* spp. OTUs in the SMT were highest in April and August with a drop in export fluxes from May to July (Fig. 3a and b).

The occurrence of 0.15 µg/L sea-ice campesterol and 0.41 µg/L sea-ice sitosterol in the studied sea-ice samples (including *Melosira arctica* aggregates) from the Amundsen Basin can be considered to support the association between sympagic biota and terrigenous biomarkers entrained in the sea-ice.

**Fig. 4.**
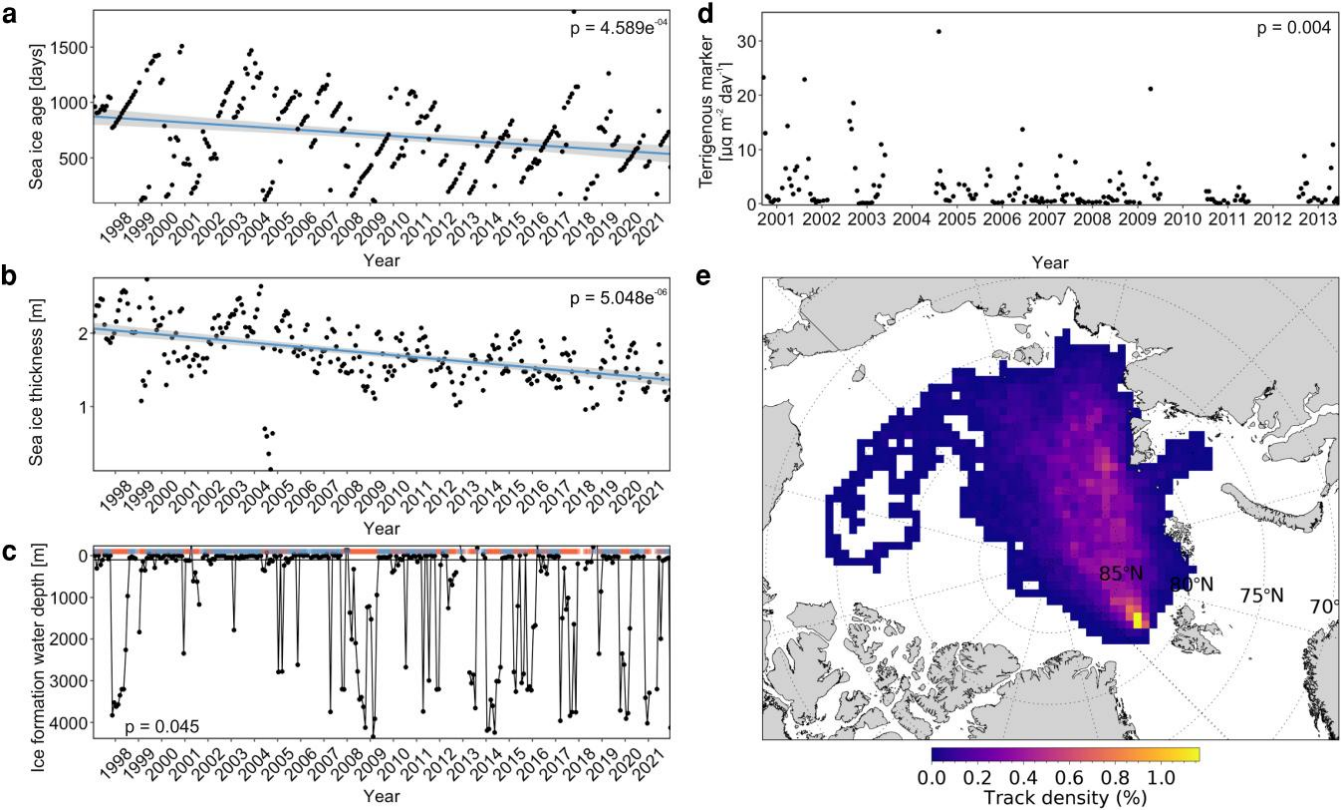
Characteristics of sea-ice exported through the Fram Strait from 1997 to 2021, derived from a sea-ice backtracking experiment and terrigenous marker fluxes at HG-IV from 2000 to 13. Panel (a) shows the average ice age of exported sea-ice. Panel (b) shows the modeled average sea-ice thickness of exported sea-ice. Panel (c) shows the water depth at the area where exported sea-ice was formed. Orange bars indicate sea-ice formation in areas with a water depth >100 m, while blue bars indicate a sea-ice formation depth in shallow regions with < 100 m depth. Panel d shows the terrigenous marker flux over the sampled period from 2000 to 2013. Note the change of *x*-axis values between panel (d) and (a–c). Panel (e) shows the areal track density of sea-ice that was exported through the Fram Strait from 1997 to 2021. Levels of statistical significance for a reduction over time in panel (a–d) were tested with a seasonal nonparametric Kendall rank correlation coefficient and are indicated by *P*-values.

The sea-ice coverage was integrated into the duration of the respective sampling periods from the sediment trap and increased from ∼0–5% in March to ∼20% in June and decreased afterwards to 0% during ice-free conditions in September (Fig. 3c). Terrigenous marker fluxes decreased significantly over the sampling period from 2000 to 2013 (Seasonal Mann–Kendall test; *P* < 0.001; Fig. 4d).

### Seafloor DNA samples

**Fig. 5.**
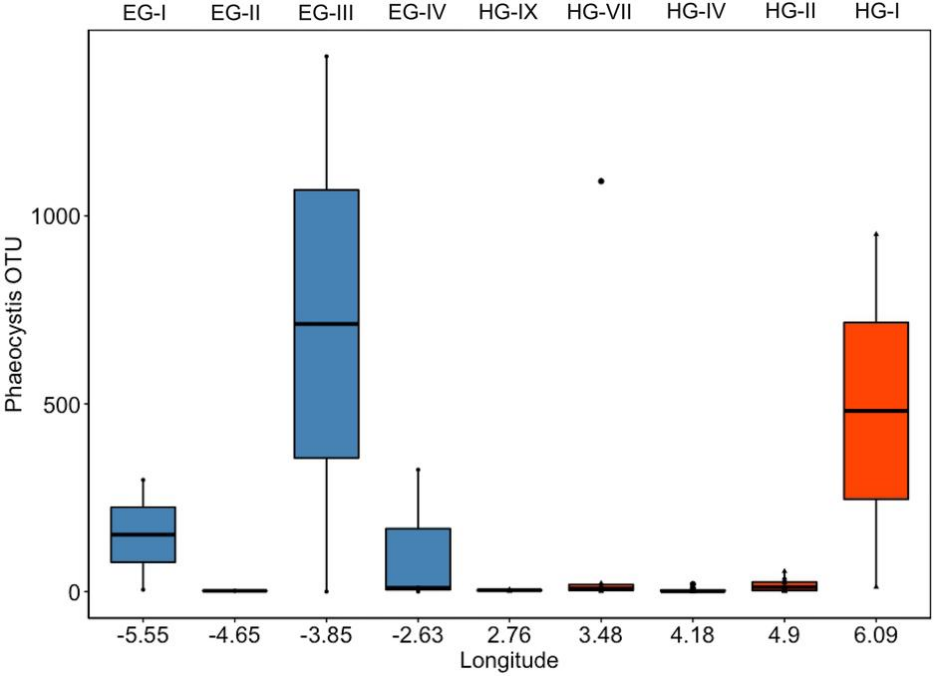
Sequence abundance of *Phaeocystis* OTUs collected from sediment samples across an east–west transect in the Fram Strait. Blue color indicates the westerly sample stations with regular sea ice cover, while red indicates easterly sample stations with rare sea ice coverage. Labels above the plot panel indicate the respective sample station.

Relative sequence abundances of *Phaeocystis* spp. were measured in deep-sea sediments collected with a multicorer along a latitudinal transect of the Fram Strait over the time period 2003–2016. Sequence abundances indicated an enhanced *Phaeocystis* spp. deposition at the ice-covered stations located in the East Greenland Current (EG stations) compared to the HG stations located in mainly ice-free Atlantic waters of the WSC (West Spitsbergen Current) (Fig. 5). However, sample availability for the EG stations was limited (*n* = 8) compared to the HG stations (*n* = 37) and showed a large variability in the sequence abundances of *Phaeocystis* spp. OTUs. Therefore, our interpretation of higher export of *Phaeocystis* spp. at the EG stations compared to the HG stations could not be verified by statistical testing.

### Gypsum scavenging experiment

**Fig. 6.**
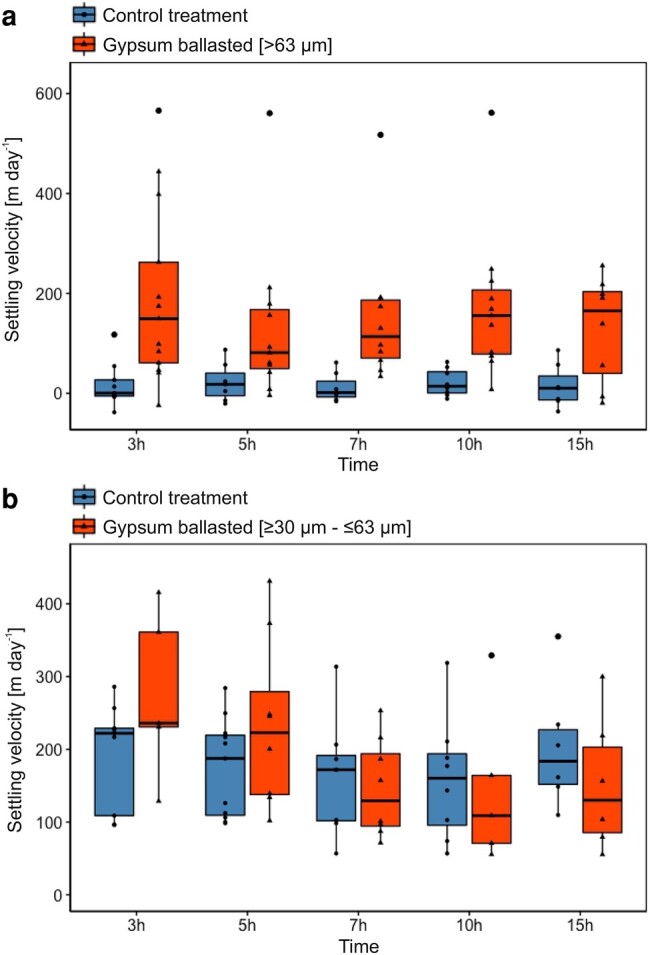
Measured settling velocities of in-situ particles over time from a ballasting experiment with and without added gypsum crystals during research expedition PS121. Gypsum crystals were added at 0 h for the ballasting treatments (red) while no gypsum was added in the control treatment (blue). Panel a compares particle settling velocities of a control treatment with particles ballasted with large gypsum crystals (>63 µm). Panel b compares particle settling velocities of a control treatment with particles ballasted with small gypsum crystals (≥30 to ≤63 µm). Settling velocities of the large-sized ballasting treatment (panel a) were significantly higher than the control treatment (ANOVA, *P* ≤ 0.05). Settling velocities of the small-sized ballasting treatment (panel b) were not elevated enough to show a significant increase over the control treatment (ANOVA, *P* ≥ 0.05).

We incubated in situ formed organic aggregates that were collected 10 m below the depth of the chlorophyll maximum layer in roller tanks with large (>63 µm) and small (>30 to <63 µm) sized gypsum crystals (collected from under sea-ice) to test how scavenging of the gypsum affected the size-specific settling velocities of formed aggregates. The scavenging experiment with large-sized gypsum crystals (>63 µm) showed a significantly higher particle settling velocity (two-way ANOVA, *P* < 0.05) over the entire 15 h of the experiment when compared to the control treatment (Fig. 6a). Further, particle settling velocities did not decrease over time, i.e. between 0 and 15 h of incubation.

In contrast, the scavenging experiment with small-sized gypsum crystals (≥30 to ≤63 µm) showed a noticeable, but not significant increase of particle settling velocities 3 and 5 h after the addition of gypsum, and decreased afterwards to similar settling velocities as the control treatment (Fig. 6b). A detailed overview of measured settling velocities is listed in Table S3.

### Sea-ice tracking

Satellite-based trajectories of sea-ice leaving Fram Strait were calculated in monthly intervals over the period from 1997 to 2021. The age of sea-ice exiting through the Fram Strait significantly decreased over the sampling period from 1997 to 2021 (seasonal Mann–Kendall test, *P* < 0.01, tau = −0.24; Fig. 4a). This is in line with the significant reduction of the modeled sea-ice thickness from 1997 to 2021 (seasonal Mann–Kendall test, *P* < 0.001, tau = −0.40; Fig. 4b). The area in which the exported sea-ice was formed significantly shifted from 1997 to 2021 from shallow shelf areas to regions with larger depths in the open Arctic Ocean (seasonal Mann–Kendall test, *P* < 0.05, tau = −0.103; Fig. 4c).

## Discussion

### POC export is enhanced in the sea-ice vicinity

POC fluxes measured over a period of 13 years during the productive season at HG-IV were significantly enhanced with sea-ice proximity of 0–40 km (*P* < 0.05, Fig. 2c) and we observed that POC flux was positively correlated to terrestrial marker flux (*P* < 0.05, Fig. 2e). The sea-ice melt and release of terrigenous material caused a ballasting of settling organic matter and resulted in elevated POC and terrestrial marker flux compared to periods with larger distance to the ice-edge, i.e. where no terrigenous material was collected by the SMT (Fig. 3a and b). Similar observations have been made in previous studies where elevated POC and ice-rafted sediment fluxes were found in the vicinity of the sea-ice edge compared to ice-free regions (11, 14). This suggests that the release of terrestrial and lithogenic material from melting sea-ice at the ice-edge can ballast organic matter, causing it to sink faster and thereby increase POC flux. It should be noted though, that an additional contribution of gypsum-mediated ballasting to enhanced POC flux cannot be verified from sediment trap samples, since any collected gypsum would dissolve before sample recovery (21). The particle ballasting is supported by direct observations of higher particle settling velocities in ice-associated regions compared to ice-free areas (14, 15). While a correlation between POC and terrigenous fluxes alone does not confirm ballasting of organic aggregates by ice-rafted material, these combined observations of elevated POC, terrigenous material flux as well as higher particle settling velocities in ice-associated areas, suggest particle ballasting as an important driver for elevated POC export at or near the ice-edge during ice-melt.

### Ballasting potential by cryogenic gypsum

We observed a temporary increase in particle settling velocities when gypsum crystals were present (Fig. 5), supporting the proposed ballasting effect of gypsum (19). However, this effect was short-lived (3–5 h) for small gypsum crystals (>30 to < 63 µm), due to the rapid dissolution in seawater and within the aggregates at positive temperatures (21). Nevertheless, small gypsum crystals could still enhance POC fluxes out of the upper water column, where organic matter is most vulnerable to attenuation due to the high zooplankton grazing (5). It should also be noted that these results are conservative, as we incubated at a temperature of 2 °C, which is comparable to Atlantic water temperatures in Fram Strait (21), whereas other Arctic waters are colder (−1 °C). Therefore, as gypsum dissolution decreases with decreasing water temperature, gypsum ballasting by small crystals would have a longer and more pronounced ballasting potential in the colder Arctic waters.

In contrast, ballasting by large gypsum crystals (>63 µm) caused a 10-fold increase in sinking velocities, which lasted until the end of the incubation (after 15 h), presumably due to their smaller surface-to-volume ratio which resulted in a slower dissolution (21). Hence, >63 µm sized gypsum crystals have the potential to enhance POC flux to the deep ocean and mediate POC deposition on the sea floor in both relatively warm Atlantic and cold Arctic waters.

Considering that we added a similar mass of small and large-sized gypsum crystals in the experiments, this implies that the ballasting effect by gypsum is largely dependent on the size distribution of gypsum crystals, i.e. the dissolution time, and not only the quantity of the gypsum crystals released during sea-ice melt. However, it should be noted that *Phaeocystis* spp. aggregates which were observed on the seafloor at >2,000 m depth, contained idiomorphic gypsum crystals (19). Due to the idiomorphic appearance, this indicates that the surrounding aggregate material protected the gypsum crystals from dissolution. Taking into account that size distributions of gypsum crystals indicate a higher relative contribution of large gypsum crystals in ≤ 2-y-old sea-ice compared to 3-y-old sea-ice (21), future predictions of more first-year sea-ice (38) may lead to a higher ballasting potential via cryogenic gypsum. In contrast to gypsum, the ballasting effect by terrigenous material is not temporarily limited by dissolution (17) and therefore, the quantity of released terrigenous material is likely the determining factor of its ballasting potential. Consequentially, particles that are ballasted by terrigenous material may have an overall higher potential to mediate deposition of POC on the seafloor, especially in the warmer Atlantic waters where sea-ice typically melts.

### *Phaeocystis* spp. export as an indicator for sea-ice mediated ballasting

To further assess potential particle ballasting from the bulk samples of the sediment trap time series, we used the export flux of the algae *Phaeocystis* spp. as an indirect indication of particle ballasting. *Phaeocystis* spp. forms colonies with large amounts of gelatinous polysaccharides, which increase buoyancy and therefore lower their sinking velocities to ∼10 m day^−1^, with reports of *Phaeocystis* spp. colonies even rising to the surface (39, 40). As a result, *Phaeocystis* spp. is usually considered to be retained within the upper 100 m of the water column and does not contribute notably to export flux without being ballasted (34–37). However, we observed a flux of *Phaeocystis* spp. to 180–340 m at the vicinity of the ice edge (Fig. 2d). This suggests that the *Phaeocystis* spp. flux must have been ballasted, which supports previous observations of the export of ballasted *Phaeocystis* spp. (13). While it should be noted that vertical mixing (35) and downwelling (12) were suggested to export *Phaeocystis* spp. to water layers ≥ 100 m, only an increase in density by ballasting was observed to export *Phaeocystis* spp. to greater depths and even deposition on the sea floor in >2,000 m (19).

To further verify the possibility of ice mediated ballasting, we analyzed sediment samples across an east–west transect along the Fram Strait for the deposition of *Phaeocystis* spp. We observed *Phaeocystis* spp. across the entire transect, with a notably enhanced deposition in the more frequently ice-associated part of the western Fram Strait (EG stations), despite the common occurrence of *Phaeocystis* spp. throughout the entire Fram Strait (Fig. S1; Refs. (13, 41, 42)). Therefore, we suggest that the efficient export and deposition of *Phaeocystis* spp. at depths >2,000 m was due to ballasting by cryogenic gypsum and/or ice rafted sediments, which were released during sea-ice melt in the Fram Strait.

### Sea-ice derived ballasting—an overlooked process in the Arctic?

The efficient export of organic matter in ice-covered regions has previously been observed as mass-sedimentation events of the cryopelagic algae *Melosira arctica* (43) and indications toward high carbon transfer efficiencies from the surface to the seafloor by benthic remineralization rates (44). The effective transport of *M. arctica* might have been increased by additional ballasting through terrigenous material. This was supported by the presence of campesterol and sitosterol in algae aggregates sampled from sea-ice leads during the same campaign where the mass sedimentation of *M. arctica* was observed. In addition, *M. arctica* has also been shown to contain cryogenic gypsum, when it is released as colonies from the sea-ice during the melting season (21). This suggests that sea-ice-mediated particle ballasting may be a common, but overlooked, driver for efficient carbon export in the Arctic.

**Fig. 7.**
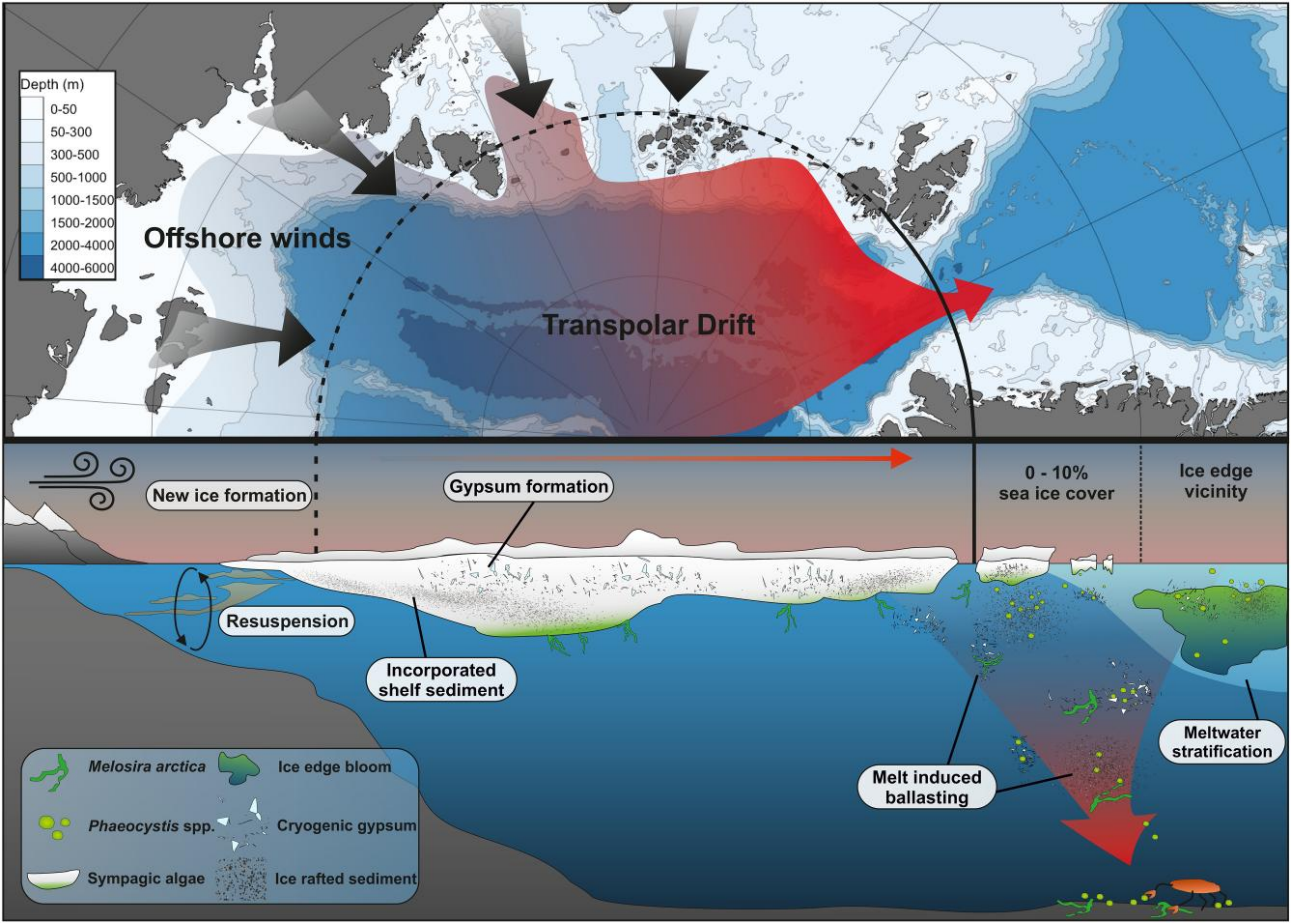
Overview schematic of the proposed process of sea-ice mediated ballasting. Newly formed sea-ice incorporates resuspended sediments over the shallow Siberian shelf and cryogenic gypsum precipitates within the highly saline brine channels. The sea-ice is transported by the transpolar drift across the Arctic Ocean and is consequentially exported through the Fram Strait where it melts. During the melt, incorporated sediments and cryogenic gypsum are released. When encountered with organic matter that forms in, under, or in the vicinity of the sea-ice, the released ice-rafted material may ballast the organic matter which increases the settling velocity which drives a more efficient drawdown of carbon to the deep ocean and supports prevailing communities with food.

The proposed processes of particle ballasting by ice-rafted material are summarized in Fig. 7. Terrigenous material is incorporated during sea-ice formation while cryogenic gypsum may form in first year ice from ion precipitation in the sea-ice brine. The ice-rafted material is transported along the Arctic Transpolar Drift and released during sea-ice melt at the outflow gateways of the Arctic Ocean, e.g. Fram Strait. When the released material encounters organic matter as it sinks through the water column, it may act as a ballasting component, which increases POC flux and therefore drives a more efficient export of carbon to the deep ocean.

Recently it has been shown that primary production in the Fram Strait is linked to the mixed layer depth (MLD) and the sea-ice edge (10). In direct proximity to the sea-ice edge, meltwater forms a strong halocline with a narrow MLD which vertically limits primary production while maintaining the phytoplankton in surface waters, thus delaying carbon export until late summer (10). In contrast, less sea-ice melt formed a deeper reaching MLD which supported a higher phytoplankton biomass that was exported earlier in the season (10). The limited carbon export during the narrow MLD regime matches well with our observations that showed that POC export is limited when sea-ice cover is ≥ 15%. However, despite a potentially limited primary production by a narrow MLD, this does not rule out a more efficient and direct export closer to the ice edge under less sea-ice cover. However, it should be noted that during the period of the observations (2017 and 2018; Ref. (10)), ballasting by terrigenous material was likely limited since the sea-ice exported through the Fram Strait was not formed on the shallow shelf and therefore did not incorporate shelf sediment during ice-formation (Fig. 4c). As a result, the formation of the MLD and release of ballasting material from melting sea-ice alter the potential for carbon export in the marginal ice zone according to the amount of sea-ice cover. The MLD promotes an enhanced amount of primary production from >20 to 0% sea-ice cover (10), while the onset of particle ballasting when sea-ice cover drops ≤ 15% leads to a more direct and efficient carbon export (Fig. 3).

### Implications of global warming on sea-ice mediated ballasting

Rising temperatures in the Laptev Sea and the central Arctic Ocean are leading to a reduction of sea-ice formation in shallow areas and thereby reduced the potential for the entrainment of terrigenous material from shallow shelf regions (Fig. 4c; Refs. (22, 23)). Coupled with the continuous reduction of long-range sea-ice transport and an earlier release of ice-rafted material in the Central Arctic (24, 45), the continuous decrease of terrigenous marker fluxes between 2000 and 2013 in the Fram Strait (Fig. 4d; Ref. (29)) suggests a decreased potential for particle ballasting via terrigenous ice-rafted material in the sea-ice vicinity of Fram Strait and hence, may reduce the export efficiency of the biological pump. As temperatures in the Arctic continue to rise, we expect that the uptake and transport of ice-rafted material and thus the effect of terrestrial ballasting will further reduce and ultimately cease when the transarctic sea-ice transport from the Siberian shelf is disrupted (Fig. 4c).

Nevertheless, there are uncertainties as to how cryogenic gypsum ballasting might impact POC flux. Increased proportions of younger and thinner sea-ice (38) were suggested to support increased primary production of sympagic algae due to higher light availability (32). Considering younger sea-ice contains higher gypsum contents than thicker older sea-ice (21), a combined effect of higher primary production and gypsum content might enhance the gypsum ballasting potential and hence, carbon export. Thus, an increase in gypsum ballasting may (over)compensate for reduced ballasting via terrigenous material.

Recent observations from the same sediment trap time-series show an increase in surface chlorophyll concentrations between 2001 and 2013 (46), while POC export in the vicinity of the ice-edge did not increase. This may indicate a decrease in the export efficiency of surface production in the Fram Strait, which could be connected to a reduction of particle ballasting vie terrigenous material in the sea-ice vicinity. It should be noted though, that it is not clear to what extent potential shifts in phytoplankton community composition may drive a reduction of the export efficiency in the Fram Strait (42, 46). In conclusion, though gypsum ballasting is projected to increase in future, it is unclear if gypsum release will compensate for ballasting via terrigenous material and lead to an increase in the quantity of Arctic carbon export, at least in the Fram Strait.

## Material and methods

### Sediment trap fluxes and sea-ice concentrations at station HG-IV

Field samplings were performed at the long-term ecological research (LTER) observatory HAUSGARTEN located in the eastern Fram Strait (Fig. 1). Flux measurements were obtained from moored SMT at station HG-IV (∼79°N; 04°E) of the observatory. Here, sinking particles including marine microorganisms from all size classes were sampled by modified automatic Kiel SMT (230 K/MT), with a sampling area of ∼0.5 m^2^, and 20 liquid-tight sampling cups (47–49). The moored long-term SMT were deployed annually from 2001 to 2013 (not every year and month were fully covered, see Table S1; see Refs. 29 and 46 for further information) between 180 and 340 m depth (except from 2009 July 20 to 2010 July 15 where the deployment depth was 80 m; see Table S1). In the years after 2013, the protocol was changed and these trap results still need quality control on their comparison to this time-series. The collection period of sampling cups lasted for 10–31 days, respectively, and shorter collection periods were set during the productive season (Table S1).

Details on sample handling and measurements of POC fluxes can be obtained from Ref. (10). Terrigenous marker fluxes as a proxy for the release of sedimentary ice rafted material included campesterol (24-methylcholest-5-en-β-ol) and sitosterol (24-ethylcholest-5-en-3β-ol) and obtained according to Ref. (49). To support the concept of these terrigenous markers in association with sea-ice derived biota, samples of different sea-ice habitats including melt ponds (with and without *Melosira arctica* aggregates), collected in the Amundsen Basin (for details, see Ref. (50)) were also analyzed for campesterol and sitosterol.

Data of sea-ice concentration and extent were obtained from NSIDC/NOAA (National Snow and Ice Data Center/National Oceanic and Atmospheric Administration) and estimated according to Ref. (10), where the sea-ice edge was defined as areas with 15% sea-ice concentration. Consequently, the distance to the ice-edge was defined as the distance from the area with 15% sea-ice concentration to the station HG-IV in central HAUSGARTEN. Sediment trap fluxes obtained during periods with an average sea-ice edge distance >150 km to station HG-IV were not considered for analysis. An overview of sediment trap fluxes and sea-ice distributions is given in Table S1.

### eDNA analyses: sampling and DNA isolation

Sediment trap samples for eDNA-analyses covered the deployment years 2001, 2006, and 2012 as well as peak productive periods between 2000 and 2013 (sample details shown in Table S1). The samples were split by a wet splitting procedure after the removal of zooplankton (swimmers) > 0.5 mm, which were manually removed under a dissecting microscope at a magnification of 20 and 50. Subsequent molecular analyses are based on 1/32 splits of the original sediment trap sample. We collected cells for DNA isolation of DNA by filtration of a split fraction from the original sample onto a 0.22-µm Sterivex-Filter (Millipore, Schwalbach, Germany). Filters were washed with sterile North Sea water (∼50 mL). Genomic DNA was isolated from the samples with the PowerWater DNA Isolation Kit (Qiagen, Hilden, Germany) according to the manufacturer’s protocol.

Benthic eDNA analyses were based on processing samples from the upper 1 cm of sediment cores collected with a TV-guided multicorer at 45 stations along an east–west transect covering 5.6° E and 6° W at ∼79° N (Table S2) between 2003 and 2016. Tip-less syringes were used to collect three smaller subsamples from the original core. Subsequent to sampling, the syringes containing the sediment were stored at −20 °C until further processing. For DNA-isolation, the first top centimeter (0–1 cm) of core in the syringe was cut off using a sterile scalpel. From each of the three subsamples, ∼0.5 g sediment was pooled, while 0.25 g of this pool was subjected to further processing. Genomic DNA was isolated using the DNeasy PowerSoil Kit (Qiagen, Hilden, Germany) following the manufacturer's protocol. DNA concentrations of both, sediment trap- and benthic samples were determined using the Quantus Fluorometer (Promega, Germany) according to the manufacturer's protocol for measuring double-stranded DNA. The resulting DNA-extracts were stored at −80 °C until further analyses.

#### Illumina sequencing of 18S rDNA

An overview of the performed sequencing analysis is outlined in Metfies et al. (51). In Illumina sequencing, a portion of the 18S rDNA containing the hypervariable V4 region was amplified using the primer set 528iF (5′-GCGGTAATTCCAGCTCC-3′) (52) and 938iR (5′-GGCAAATGCTTTCGC-3′). All PCRs had a final volume of 25 µL, consisting of 12.5 µL mastermix (KAPA HiFi HotStart ReadyMix, KAPABiosystems, Roche), 2.5 µL of each primer (1 µmol), and 2.5 µL genomic DNA (∼5 ng/µL). PCR amplification occurred in a thermal cycler (Eppendorf, Germany), initiating with an initial denaturation (95 °C, 3 min), followed by 25 cycles of denaturation (95 °C, 30 s), annealing (55 °C, 30 s), and extension (72 °C, 30 s), with a single final extension (72 °C, 5 min). Subsequently, PCR products were purified from a 1% [w/v] agarose gel using the AMPure XP PCR purification kit (Beckman Coulter, Inc., USA), following the manufacturer’s protocol. After purifying the 18S rDNA fragment, DNA concentrations of the samples were determined using the Quantus Fluorometer (Promega, USA).

Attachment of Indices and Sequencing Adapters: Indices and sequencing adapters from the Nextera XT Index Kit (Illumina, USA) were attached through Index PCR. The PCR had a final volume of 50 µL, including 25 µL of KAPA HIFI Mix (Kapa Biosystems, Roche, Germany), 5 µL of each Nextera XT Index Primer [1 µmol/L], 5 µL DNA-template [∼5 ng], and 10 µL PCR grade water. PCR amplification occurred in a thermal cycler (Eppendorf, Germany), beginning with an initial denaturation (95 °C, 3 min), followed by eight cycles of denaturation (95 °C, 30 s), annealing (55 °C, 30 s), and extension (72 °C, 30 s), with a single final extension (72 °C, 5 min). Before quantifying the PCR products with the Quantus Fluorometer (Promega, USA) for sequencing with the MiSeq Sequencer (Illumina, USA), the final library underwent cleanup using the AMPure XP PCR purification kit (Beckman Coulter, Inc., USA). Sequencing of DNA fragments was carried out using the MiSeq Reagent Kit V3 (2×300 bp), following the manufacturer's protocol (Illumina, USA). Raw sequences generated in this study have been deposited in the European Nucleotide Archive (ENA) with accession number xxx (to be provided upon manuscript acceptance).

#### Sequence analyses

Raw reads underwent quality trimming with Trimmomatic (53). This involved scanning reads with a four-base wide sliding window and cutting when the average quality dropped below 15. For merging paired-end reads, the script join-paired-ends within the open-source bioinformatics pipeline QIIME v.1.8.0 (54) was utilized, requiring a minimum read overlap of 20 bases. Further analyses were performed using QIIME v.1.8.0 (54). In summary, reads were quality-filtered according to recommended settings (55). Only sequences fully matching the primer sequences at the beginning and end, respectively, and falling between 200 and 500 bp in length were further processed. For chimera detection and clustering of sequences into OTUs, the QIIME workflow “usearch.qf” was employed, incorporating the UCHIME algorithem (56). Preclustered sequences were examined for chimeras (de novo and with Silva 119 SSU Ref NR). The remaining sequence set was clustered (de novo) into OTUs with a similarity threshold of 98%. Taxonomy classification of OTUs was performed using the QIIME default sequence classifier algorithem UCLUST (57). Normalization, analyses, and visualization of sequence data were conducted in R (R Development Core Team, 2008). The sequence dataset was normalized to the lowest number of OTUs in the study using the rarefy function from the Vegan package. 18S-sequences of *Phaeocystis* spp. OTUs are published in GenBank, and sequence abundances in the samples are published in PANGAEA. Accession numbers will be provided upon manuscript acceptance.

### Gypsum scavenging experiment

To study the ballasting effect of cryogenic gypsum crystals on particle settling, we used a modified approach of the “scavenging experiment” from Ref. (18) where we observed settling velocities of in-situ marine snow particles which were encountered with gypsum crystals. Cryogenic gypsum crystals used in the experiments were collected in-situ during research expedition PS106 1/2 in 2017 (see Ref. (21) for further details). Experiments were performed during research expedition PS121 in 2019.

For the experiment, in-situ marine snow aggregates were collected with a marine snow catcher (MSC). The collection depth was set 10 m below the chlorophyll maximum layer, which was detected from fluorescence readings and ranged between 40 and 60 m. Captured marine snow aggregates were let to settle for 8 h within the MSC, before the overlying water was gently drained. Collected aggregates were evenly divided into six roller tanks (1.15 L tank^−1^) filled with filtered seawater from the MSC, of which one was used as a control while a gypsum suspension was added to the remaining five tanks. A total of four experiments were carried out in a temperature-controlled laboratory set at 2 °C, with two utilizing a gypsum suspension with crystals ≥ 30 to ≤ 63 µm (24.32 and 20.06 mg L^−1^) and two with crystals ≥ 63 µm (21.12 and 23.33 mg L^−1^). The gypsum crystals were suspended in 50 mL filtered seawater from which 10 mL was added to each roller tank, respectively. The gypsum-treated roller tanks were designated to one of five time points, respectively (3, 5, 7, 10, and 15 h), and placed on a roller table set at 3 RPM. For each time point, video recordings of the designated tank and the control tank were taken (Sony alpha a7II; Zeiss Loxia 50mm f2.2). Measurements of particle size and settling velocity were obtained from the captured video recordings according to Ref. “(58)”. As scavenging experiments were conducted one after another, the regions where in-situ aggregates were collected varied between scavenging experiments with ≥ 30 to ≤63 and ≥ 63 µm sized gypsum crystals. As a result, aggregate settling velocities of the respective control groups for the experiments with ≥ 30 to ≤63 and ≥ 63 µm sized gypsum crystals varied. Measured settling velocities from roller tank experiments with gypsum crystals ≥ 30 to ≤63 and crystals ≥ 63 µm were grouped, respectively, for further analysis.

### Sea-ice tracking

In order to determine trajectories and origin of sea-ice passing the sediment trap position at 79°N/4.3°E, we applied a Lagrangian approach called ICETrack. The approach traces sea-ice backward in time using a combination of low-resolution satellite and atmospheric reanalysis data products. So far, ICETrack has been used in a number of publications to examine sea-ice sources, pathways, thickness changes, and atmospheric processes acting on the ice cover (59–61).

In summary, IceTrack uses a combination of the following three different ice drift products for the tracking of sea-ice: (i) motion estimates provided by the Centre for Satellite Exploitation and Research (CERSAT; 61), (ii) the OSI-405-c motion product from the Ocean and Sea Ice Satellite Application Facility (OSI SAF; 62), and (iii) Polar Pathfinder Daily Motion Vectors (v.4) from the National Snow and Ice Data Center (NSIDC; 63). The IceTrack algorithm first checks for the availability of CERSAT motion data, since CERSAT provides the most consistent time series of motion vectors starting from 1991 to present and has shown reliable performance (64). During the summer months (June–July), when drift estimates from CERSAT are missing, motion information is bridged with the OSI SAF product (2012 to present). Before 2012, or if no valid OSI SAF motion vector is available within the search range, NSIDC data are applied. Along the track, several additional parameters from various satellite and reanalysis products are derived. For example, sea-ice concentration is retrieved from a CERSAT product (65), while the thermodynamic ice thickness was calculated by means of NCEP (National Centers for Environmental Prediction) reanalysis 2m-air temperature data (66) following Ref. “(67)”. The model was tested and applied in Ref. “(61)”.

The reconstruction of sea-ice trajectories works as follows: sea-ice is traced backward in time every month starting on the first of each month (1997–2021). Tracking is discontinued if sea-ice concentration at a specific location along the trajectory drops below 25%, which the algorithm defines as the position at which the ice is formed. Since the study of Ref. (68) indicated a limited performance of IceTrack in the Fram Strait, tracking performed in this study is initiated at a position located further north. This position (83°N/11°E) was chosen to be in the catchment area of the sediment trap, which was determined in a separate tracking experiment.

### Statistical analysis

Statistical analysis of long-term trends in sediment trap fluxes and environmental data was performed with a seasonal nonparametric Kendall rank correlation coefficient. The monthly difference of export fluxes measured in a distance of 0–40 km and 40–80 km from the ice edge were tested with a Wilcoxon rank sum test. The correlation between POC and terrigenous marker flux measured with the SMT was tested with a Spearman rank correlation.

For the gypsum ballasting experiments, settling velocities where Log_10_ transformed to assure a normal distribution after which a two-way ANOVA and Tukey test were performed to test for difference in settling velocity between control and gypsum added treatments. All statistical testing was performed in R (R Core Team 2021).

## Supplementary Material

pgae081_Supplementary_Data

## Data Availability

Data used in this study are made available at PANGEA. 18S-sequences of *Phaeocystis* spp. OTUs are published in Genbank and sequence abundances in the samples are published in PANGAEA. Accession numbers will be provided subsequent to acceptance of the manuscript.
