# Supplementary material for: Release of ballast material during sea-ice melt enhances carbon export in the Arctic Ocean
Source: PNAS Nexus. 2024 Feb 17;3(4):pgae081. doi: 10.1093/pnasnexus/pgae081 (PMC10978062; doi:10.1093/pnasnexus/pgae081)
Supplement: pgae081_Supplementary_Data [file pgae081_supplementary_data.docx]

Supplementary Materials for:

**Release of ballast material during sea-ice melt enhances carbon export in the Arctic Ocean**

Steffen Swoboda^1*^, Thomas Krumpen^2^, Eva-Maria Nöthig^2^, Katja Metfies^2^, Simon Ramondenc^1,2^, Jutta Wollenburg^2^, Kirsten Fahl^2^, Ilka Peeken^2^, Morten Iversen^1,2*^

*Corresponding author. Email: [sswoboda@marum.de](mailto:sswoboda@marum.de) ; [morten.iversen@awi.de](mailto:morten.iversen@awi.de)

**This file includes:**

Tables S1 to S3

Table S1.

| Research expedition | Mooring location | | Deployment  depth (m) | Sampling interval |  | POC | Terrigenous  marker | Phaeocystis | Ice edge distance | Ice concentration |
| --- | --- | --- | --- | --- | --- | --- | --- | --- | --- | --- |
|  | Latitude  N | Longitude  W |  | date start | date end | mg m^-2^ d^-1^ | µg m^-2^ d^-1^ | OTU | km | % |
| PS57 | 79°01.7 | 04°20.86 | 280 | 31/08/2000 | 15/09/2000 | 26.1 | 88.8 | 0 | 1 | 76 |
|  |  |  |  | 15/09/2000 | 30/09/2000 | 12.1 | 23.3 | NA | 58 | 0 |
|  |  |  |  | 14/03/2001 | 29/03/2001 | 9.1 | 6.5 | 0 | 41 | 0 |
|  |  |  |  | 29/03/2001 | 14/04/2001 | 8.9 | 14.3 | 0 | 24 | 5 |
|  |  |  |  | 14/04/2001 | 29/04/2001 | 4.8 | 4.6 | 0 | 43 | 0 |
|  |  |  |  | 29/04/2001 | 14/05/2001 | 5.5 | 3.3 | 0 | 51 | 0 |
|  |  |  |  | 14/05/2001 | 29/05/2001 | 3.4 | 1.8 | 0 | 87 | 0 |
|  |  |  |  | 29/05/2001 | 14/06/2001 | 3.7 | 6.2 | 0 | 50 | 0 |
|  |  |  |  | 14/06/2001 | 29/06/2001 | 2.1 | 6.9 | 0 | 52 | 1 |
|  |  |  |  | 29/06/2001 | 14/07/2001 | 3.3 | 2.6 | 0 | 13 | 10 |
| PS59 | 79°01.5 | 04°21,3 | 260 | 14/08/2001 | 31/08/2001 | 17.7 | 22.9 | 0 | 38 | 0 |
|  |  |  |  | 31/08/2001 | 15/09/2001 | 6.1 | 4.9 | 0 | 83 | 0 |
|  |  |  |  | 15/09/2001 | 30/09/2001 | 7.8 | 8.3 | 0 | 102 | 0 |
| PS62 | 79°01.04 | 04°19.77 | 260 | 14/08/2002 | 29/08/2002 | 7.3 | 15.2 | NA | 144 | 0 |
|  |  |  |  | 29/08/2002 | 13/09/2002 | 7.0 | 13.8 | NA | 138 | 0 |
|  |  |  |  | 13/09/2002 | 28/09/2002 | 8.6 | 18.6 | NA | 102 | 0 |
|  |  |  |  | 28/09/2002 | 13/10/2002 | 2.5 | 6.6 | NA | 71 | 0 |
|  |  |  |  | 12/03/2003 | 27/03/2003 | 3.1 | 1.3 | NA | 54 | 0 |
|  |  |  |  | 27/03/2003 | 11/04/2003 | 6.8 | 1.7 | NA | 22 | 7 |
|  |  |  |  | 11/04/2003 | 26/04/2003 | 7.5 | 3.3 | NA | 16 | 9 |
| PS64 | 78°59.95 | 04°27.44 | 340 | 26/04/2003 | 11/05/2003 | 15.3 | 10.9 | 40 | 12 | 10 |
|  |  |  |  | 11/05/2003 | 26/05/2003 | 7.3 | 5.2 | NA | 18 | 7 |
|  |  |  |  | 26/05/2003 | 10/06/2003 | 12.5 | 9.0 | NA | 3 | 27 |
| PS66 | 79°00.99 | 04°20.62 | 280 | 15/07/2004 | 22/07/2004 | 1.9 | 2.0 | NA | 52 | 0 |
|  |  |  |  | 22/07/2004 | 29/07/2004 | 7.2 | 3.7 | NA | 50 | 0 |
|  |  |  |  | 29/07/2004 | 05/08/2004 | 49.4 | 67.9 | 58 | 105 | 0 |
|  |  |  |  | 05/08/2004 | 15/08/2004 | 42.0 | 31.7 | 30 | 216 | 0 |
|  |  |  |  | 15/08/2004 | 31/08/2004 | 13.9 | 6.1 | NA | 271 | 0 |
|  |  |  |  | 31/08/2004 | 15/09/2004 | 12.3 | 3.5 | NA | 246 | 0 |
|  |  |  |  | 15/09/2004 | 30/09/2004 | 6.5 | 3.0 | NA | 175 | 0 |
|  |  |  |  | 28/02/2005 | 31/03/2005 | 7.6 | 3.5 | 149 | 54 | 0 |
|  |  |  |  | 31/03/2005 | 15/04/2005 | 4.8 | 2.6 | NA | 69 | 0 |
|  |  |  |  | 15/04/2005 | 30/04/2005 | 3.6 | 1.6 | NA | 35 | 0 |
|  |  |  |  | 30/04/2005 | 15/05/2005 | 3.1 | 1.0 | NA | 69 | 0 |
|  |  |  |  | 15/05/2005 | 31/05/2005 | 2.2 | 1.0 | NA | 69 | 0 |
|  |  |  |  | 31/05/2005 | 15/06/2005 | 3.7 | 1.7 | NA | 49 | 0 |
| PS68 | 79°01.00 | 04°20,62 | 179 | 25/08/2005 | 05/09/2005 | 9.6 | 3.5 | NA | 84 | 0 |
|  |  |  |  | 05/09/2005 | 25/09/2005 | 15.9 | 6.4 | 15 | 61 | 0 |
|  |  |  |  | 25/09/2005 | 15/10/2005 | 8.1 | 5.1 | NA | 24 | 4 |
|  |  |  |  | 28/02/2006 | 30/03/2006 | 4.7 | 1.6 | 0 | 72 | 0 |
|  |  |  |  | 30/03/2006 | 14/04/2006 | 2.8 | 0.7 | 0 | 66 | 0 |
|  |  |  |  | 14/04/2006 | 29/04/2006 | 6.5 | 2.0 | 0 | 65 | 0 |
|  |  |  |  | 29/04/2006 | 14/05/2006 | 13.5 | 4.9 | 19 | 39 | 2 |
|  |  |  |  | 14/05/2006 | 29/05/2006 | 5.6 | 2.8 | 0 | 78 | 0 |
|  |  |  |  | 29/05/2006 | 13/06/2006 | 11.4 | 7.2 | 11 | 56 | 0 |
|  |  |  |  | 13/06/2006 | 28/06/2006 | 23.9 | 13.7 | 0 | 94 | 0 |
|  |  |  |  | 28/06/2006 | 13/07/2006 | 0.8 | 0.3 | 0 | 60 | 0 |
|  |  |  |  | 13/07/2006 | 20/07/2006 | 1.9 | 1.4 | 0 | 65 | 0 |
| MSM2 | 79°00,82 | 04° 20.5 | 230 | 26/08/2006 | 01/09/2006 | 1.0 | 0.8 | 0 | 94 | 0 |
|  |  |  |  | 01/09/2006 | 07/09/2006 | 1.2 | 0.6 | 0 | 73 | 0 |
|  |  |  |  | 07/09/2006 | 15/09/2006 | 3.0 | 0.6 | 0 | 79 | 0 |
|  |  |  |  | 15/09/2006 | 22/09/2006 | 4.7 | 0.6 | 16 | 75 | 0 |
|  |  |  |  | 22/09/2006 | 30/09/2006 | 3.3 | 0.6 | 0 | 50 | 0 |
|  |  |  |  | 28/02/2007 | 15/03/2007 | 2.5 | 0.5 | NA | 43 | 0 |
|  |  |  |  | 15/03/2007 | 31/03/2007 | 16.6 | 5.2 | NA | 49 | 0 |
|  |  |  |  | 31/03/2007 | 15/04/2007 | 17.8 | 3.0 | NA | 36 | 1 |
|  |  |  |  | 15/04/2007 | 30/04/2007 | 33.3 | 8.8 | 22 | 14 | 8 |
|  |  |  |  | 30/04/2007 | 10/05/2007 | 50.3 | 0.8 | NA | 24 | 5 |
|  |  |  |  | 10/05/2007 | 20/05/2007 | 7.1 | 1.5 | NA | 42 | 0 |
|  |  |  |  | 20/05/2007 | 06/06/2007 | 2.4 | 0.4 | NA | 16 | 8 |
|  |  |  |  | 06/06/2007 | 20/06/2007 | 4.4 | 1.7 | NA | 26 | 5 |
| PS70 | 79°00,82 | 04° 20,62 | 190 | 23/07/2007 | 15/08/2007 | 6.3 | 1.8 | NA | 55 | 0 |
|  |  |  |  | 15/08/2007 | 31/08/2007 | 15.6 | 7.7 | 74 | 19 | 6 |
|  |  |  |  | 31/08/2007 | 10/09/2007 | 4.6 | 0.7 | NA | 30 | 2 |
|  |  |  |  | 10/09/2007 | 20/09/2007 | 1.5 | 0.1 | NA | 38 | 1 |
|  |  |  |  | 20/09/2007 | 30/09/2007 | 3.4 | 0.9 | NA | 21 | 5 |
|  |  |  |  | 29/02/2008 | 31/03/2008 | 5.1 | 0.4 | NA | 53 | 0 |
|  |  |  |  | 31/03/2008 | 15/04/2008 | 23.5 | 1.3 | 103 | 33 | 0 |
|  |  |  |  | 15/04/2008 | 30/04/2008 | 11.0 | 0.2 | NA | 1 | 29 |
|  |  |  |  | 30/04/2008 | 10/05/2008 | 14.5 | 0.0 | NA | 0 | 43 |
|  |  |  |  | 10/05/2008 | 20/05/2008 | 7.0 | 1.3 | NA | 2 | 30 |
|  |  |  |  | 20/05/2008 | 31/05/2008 | 10.1 | 4.2 | NA | 0 | 38 |
|  |  |  |  | 31/05/2008 | 15/06/2008 | 8.9 | 1.0 | NA | 0 | 36 |
|  |  |  |  | 15/06/2008 | 30/06/2008 | 2.4 | 0.6 | NA | 1 | 43 |
| PS72 | 79°00,4 | 04° 20,0 | 196 | 17/07/2008 | 31/07/2008 | 5.6 | 0.7 | NA | 7 | 17 |
|  |  |  |  | 31/07/2008 | 10/08/2008 | 16.9 | 2.8 | NA | 2 | 20 |
|  |  |  |  | 10/08/2008 | 20/08/2008 | 12.9 | 5.8 | NA | 15 | 6 |
|  |  |  |  | 20/08/2008 | 31/08/2008 | 30.0 | 39.6 | 101 | 19 | 4 |
|  |  |  |  | 31/08/2008 | 15/09/2008 | 8.5 | 3.5 | NA | 28 | 0 |
|  |  |  |  | 15/09/2008 | 30/09/2008 | 5.3 | 1.9 | NA | 31 | 1 |
|  |  |  |  | 28/02/2009 | 31/03/2009 | 9.3 | 5.0 | NA | 52 | 0 |
|  |  |  |  | 31/03/2009 | 15/04/2009 | 22.7 | 7.4 | 84 | 20 | 3 |
|  |  |  |  | 15/04/2009 | 30/04/2009 | 20.3 | 21.2 | 115 | 0 | 31 |
|  |  |  |  | 30/04/2009 | 15/05/2009 | 12.1 | 3.7 | NA | 24 | 4 |
|  |  |  |  | 15/05/2009 | 31/05/2009 | 4.6 | 1.6 | NA | 11 | 12 |
|  |  |  |  | 31/05/2009 | 15/06/2009 | 9.3 | 4.7 | NA | 17 | 9 |
|  |  |  |  | 15/06/2009 | 30/06/2009 | 4.3 | 1.0 | NA | 29 | 2 |
|  |  |  |  | 30/06/2009 | 15/07/2009 | 3.6 | 1.8 | NA | 13 | 10 |
|  |  |  |  | 15/07/2009 | 22/07/2009 | 4.5 | NA | NA | 21 | 3 |
| PS74 | 79°00,43 | 04° 20,05 | 80 | 20/07/2009 | 31/07/2009 | 25.1 | NA | NA | 44 | 0 |
|  |  |  |  | 31/07/2009 | 10/08/2009 | 42.2 | NA | 46 | 36 | 0 |
|  |  |  |  | 10/08/2009 | 20/08/2009 | 46.7 | NA | 230 | 39 | 0 |
|  |  |  |  | 20/08/2009 | 31/08/2009 | 19.5 | NA | NA | 59 | 0 |
|  |  |  |  | 31/08/2009 | 15/09/2009 | 16.7 | NA | NA | 68 | 0 |
|  |  |  |  | 15/09/2009 | 30/09/2009 | 8.0 | NA | NA | 85 | 0 |
|  |  |  |  | 28/02/2010 | 15/03/2010 | 4.1 | NA | NA | 75 | 0 |
|  |  |  |  | 15/03/2010 | 31/03/2010 | 1.9 | NA | NA | 54 | 0 |
|  |  |  |  | 31/03/2010 | 15/04/2010 | 11.8 | NA | NA | 91 | 0 |
|  |  |  |  | 15/04/2010 | 30/04/2010 | 11.8 | NA | NA | 65 | 0 |
|  |  |  |  | 30/04/2010 | 15/05/2010 | 10.1 | NA | NA | 67 | 0 |
|  |  |  |  | 15/05/2010 | 31/05/2010 | 26.0 | NA | NA | 77 | 0 |
|  |  |  |  | 31/05/2010 | 15/06/2010 | 22.4 | NA | NA | 60 | 0 |
|  |  |  |  | 15/06/2010 | 30/06/2010 | 29.3 | NA | 27 | 48 | 0 |
| PS76 | 79° 00.41 | 04°19.90 | 200 | 10/07/2010 | 20/07/2010 | 18.2 | 2.3 | NA | 59 | 0 |
|  |  |  |  | 20/07/2010 | 31/07/2010 | 11.0 | 0.9 | NA | 83 | 0 |
|  |  |  |  | 31/07/2010 | 15/08/2010 | 7.7 | 2.3 | NA | 92 | 0 |
|  |  |  |  | 15/08/2010 | 31/08/2010 | 8.2 | 0.9 | NA | 76 | 0 |
|  |  |  |  | 31/08/2010 | 15/09/2010 | 8.7 | 0.9 | NA | 41 | 0 |
|  |  |  |  | 15/09/2010 | 30/09/2010 | 6.0 | 0.4 | NA | 75 | 0 |
|  |  |  |  | 28/02/2011 | 15/03/2011 | 3.5 | 0.6 | NA | 68 | 0 |
|  |  |  |  | 15/03/2011 | 31/03/2011 | 7.8 | 1.5 | NA | 31 | 3 |
|  |  |  |  | 31/03/2011 | 15/04/2011 | 9.3 | 3.1 | NA | 17 | 7 |
|  |  |  |  | 15/04/2011 | 30/04/2011 | 8.2 | 0.7 | NA | 53 | 0 |
|  |  |  |  | 30/04/2011 | 10/05/2011 | 10.0 | 0.9 | 45 | 40 | 0 |
|  |  |  |  | 10/05/2011 | 20/05/2011 | 9.9 | 1.6 | NA | 31 | 1 |
|  |  |  |  | 20/05/2011 | 31/05/2011 | 5.8 | 0.4 | NA | 58 | 0 |
|  |  |  |  | 31/05/2011 | 15/06/2011 | 4.4 | 0.3 | NA | 25 | 3 |
|  |  |  |  | 15/06/2011 | 30/06/2011 | 5.6 | 0.4 | NA | 11 | 9 |
| PS78 | 79° 00.42 | 04°19.90 | 200 | 01/08/2011 | 15/08/2011 | 13.9 | NA | NA | 39 | 1 |
|  |  |  |  | 15/08/2011 | 31/08/2011 | 11.5 | NA | NA | 97 | 0 |
|  |  |  |  | 31/08/2011 | 10/09/2011 | 17.4 | NA | 315 | 109 | 0 |
|  |  |  |  | 10/09/2011 | 30/09/2011 | 7.6 | NA | NA | 108 | 0 |
|  |  |  |  | 28/02/2012 | 31/03/2012 | 5.7 | NA | 0 | 82 | 0 |
|  |  |  |  | 31/03/2012 | 15/04/2012 | 7.8 | NA | 0 | 66 | 0 |
|  |  |  |  | 15/04/2012 | 30/04/2012 | 18.0 | NA | 0 | 45 | 0 |
|  |  |  |  | 30/04/2012 | 10/05/2012 | 24.2 | NA | 8 | 36 | 0 |
|  |  |  |  | 10/05/2012 | 20/05/2012 | 7.5 | NA | 0 | 71 | 0 |
|  |  |  |  | 20/05/2012 | 31/05/2012 | 4.4 | NA | 0 | 62 | 0 |
|  |  |  |  | 31/05/2012 | 10/06/2012 | 19.0 | NA | 0 | 35 | 0 |
|  |  |  |  | 10/06/2012 | 20/06/2012 | 9.8 | NA | 0 | 19 | 6 |
|  |  |  |  | 20/06/2012 | 30/06/2012 | 7.4 | NA | 0 | 8 | 16 |
|  |  |  |  | 30/06/2012 | 15/07/2012 | 3.5 | NA | 8 | 58 | 0 |
| PS80 | 79° 00.43 | 04°19.78 | 205 | 29/07/2012 | 15/08/2012 | 5.9 | 1.3 | 49 | 30 | 1 |
|  |  |  |  | 15/08/2012 | 31/08/2012 | 13.1 | 2.8 | 151 | 42 | 4 |
|  |  |  |  | 31/08/2012 | 10/09/2012 | 15.8 | 1.9 | 43 | 84 | 0 |
|  |  |  |  | 10/09/2012 | 20/09/2012 | 12.5 | 8.8 | 0 | 72 | 0 |
|  |  |  |  | 20/09/2012 | 30/09/2012 | 9.9 | 3.8 | NA | 61 | 0 |
|  |  |  |  | 28/02/2013 | 31/03/2013 | 2.1 | 0.7 | NA | 44 | 0 |
|  |  |  |  | 31/03/2013 | 15/04/2013 | 7.3 | 3.0 | NA | 24 | 3 |
|  |  |  |  | 15/04/2013 | 30/04/2013 | 20.6 | 6.6 | NA | 29 | 1 |
|  |  |  |  | 30/04/2013 | 10/05/2013 | 18.7 | 10.9 | NA | 33 | 0 |
|  |  |  |  | 10/05/2013 | 20/05/2013 | 2.7 | 0.3 | NA | 23 | 0 |
|  |  |  |  | 20/05/2013 | 31/05/2013 | 2.8 | 0.6 | NA | 40 | 2 |
|  |  |  |  | 31/05/2013 | 10/06/2013 | 3.1 | 1.0 | NA | 16 | 12 |
|  |  |  |  | 10/06/2013 | 20/06/2013 | 2.0 | 0.2 | NA | 0 | 33 |
|  |  |  |  | 20/06/2013 | 30/06/2013 | 3.0 | 1.0 | NA | 1 | 26 |

**Table S1.** **List of particulate organic carbon (POC), terrigenous marker and Phaeocystis operational taxonomic unit (OTU) fluxes obtained from moored sediment trap deployments at station HG-IV in Fram Strait.** Research expeditions indicate the respective expedition where the mooring was deployed, with “PS” indicating research vessel *Polarstern* and “MSM” indicating research vessel *Maria S. Merian*. The mooring location is shown by the degrees of latitude and longitude upon deployment. Sediment traps consist of a rotating carousel of 20 sample cups which individually collected export fluxes for a designated time period. The start and end of each collection period is listed as the date start and date end respectively. Values of the distance of the sea ice edge to the mooring location are listed for each individual sampling interval. Values of sea ice cover above the mooring location are listed for each individual sampling interval.

Table S2.

| Station | Year | Longitude E & W | Latitude N | *Phaeocystis* spp. OTU |
| --- | --- | --- | --- | --- |
| EG1 | 2014 | -5.55 | 79.00 | 298 |
| EG1 | 2016 | -5.55 | 79.00 | 5 |
| EG2 | 2014 | -4.65 | 79.00 | 2 |
| EG3 | 2014 | -3.85 | 79.00 | 1425 |
| EG3 | 2016 | -3.85 | 79.00 | 0 |
| EG4 | 2014 | -2.63 | 79.00 | 10 |
| EG4 | 2015 | -2.63 | 79.00 | 325 |
| EG4 | 2016 | -2.63 | 79.00 | 0 |
| HG9 | 2003 | 2.76 | 79.14 | 1 |
| HG9 | 2004 | 2.76 | 79.14 | 4 |
| HG9 | 2015 | 2.76 | 79.14 | 5 |
| HG7 | 2004 | 3.48 | 79.06 | 1092 |
| HG7 | 2006 | 3.48 | 79.06 | 8 |
| HG7 | 2007 | 3.48 | 79.06 | 15 |
| HG7 | 2008 | 3.48 | 79.06 | 5 |
| HG7 | 2011 | 3.48 | 79.06 | 22 |
| HG7 | 2012 | 3.48 | 79.06 | 2 |
| HG7 | 2016 | 3.48 | 79.06 | 1 |
| HG4 | 2004 | 4.18 | 79.07 | 1 |
| HG4 | 2007 | 4.18 | 79.07 | 20 |
| HG4 | 2008 | 4.18 | 79.07 | 9 |
| HG4 | 2009 | 4.18 | 79.07 | 1 |
| HG4 | 2010 | 4.18 | 79.07 | 1 |
| HG4 | 2012 | 4.18 | 79.07 | 4 |
| HG4 | 2013 | 4.18 | 79.07 | 1 |
| HG4 | 2014 | 4.18 | 79.07 | 4 |
| HG4 | 2015 | 4.18 | 79.07 | 0 |
| HG4 | 2016 | 4.18 | 79.07 | 0 |
| HG2 | 2003 | 4.90 | 79.13 | 2 |
| HG2 | 2005 | 4.90 | 79.13 | 30 |
| HG2 | 2005 | 4.90 | 79.13 | 14 |
| HG2 | 2006 | 4.90 | 79.13 | 24 |
| HG2 | 2008 | 4.90 | 79.13 | 2 |
| HG2 | 2009 | 4.90 | 79.13 | 10 |
| HG2 | 2010 | 4.90 | 79.13 | 3 |
| HG2 | 2011 | 4.90 | 79.13 | 36 |
| HG2 | 2013 | 4.90 | 79.13 | 1 |
| HG2 | 2014 | 4.90 | 79.13 | 3 |
| HG2 | 2015 | 4.90 | 79.13 | 14 |
| HG2 | 2016 | 4.90 | 79.13 | 54 |
| HG1 | 2004 | 6.09 | 79.13 | 12 |
| HG1 | 2015 | 6.09 | 79.13 | 951 |

**Table S2. List of OTUs from *Phaeocystis* spp. measured in sediment samples derived from an east-west transect across Fram Strait.** The station name is listed along with the location in decimal degrees longitude and latitude and the year samples where obtained.

Table S3.

| Gypsum ballasting size | Control | | Ballasted | | Time |
| --- | --- | --- | --- | --- | --- |
|  | Settling velocity (m day^-1^) | n | Settling velocity (m day^-1^) | n |  |
| >63 µm | 18 ± 45 | 9 | 173 ± 185 | 14 | 3h |
| >63 µm | 22 ± 39 | 7 | 131 ± 158 | 11 | 5h |
| >63 µm | 12 ± 29 | 7 | 153 ± 141 | 10 | 7h |
| >63 µm | 21 ± 27 | 8 | 174 ± 148 | 11 | 10h |
| >63 µm | 5 ± 49 | 8 | 129 ± 106 | 8 | 15h |
| ≥30 µm - ≤63 µm | 193 ± 73 | 9 | 274 ± 114 | 5 | 3h |
| ≥30 µm - ≤63 µm | 174 ± 67 | 11 | 234 ± 117 | 8 | 5h |
| ≥30 µm - ≤63 µm | 164 ± 80 | 8 | 146 ± 67 | 8 | 7h |
| ≥30 µm - ≤63 µm | 159 ± 85 | 8 | 146 ± 111 | 5 | 10h |
| ≥30 µm - ≤63 µm | 202 ± 87 | 6 | 152 ± 93 | 6 | 15h |

**Table S3. List of measured particle settling velocities from control treatments and gypsum ballasted treatments during a ballasting experiment.** The gypsum ballasting size indicates the size range of gypsum crystals used for the ballasting experiments. Settling velocities show the average settling velocity and standard deviation measured over the course of the experiments, while n indicates the number of particles measured.
